# *Anopheles darlingi* polytene chromosomes: revised maps including newly described inversions and evidence for population structure in Manaus

**DOI:** 10.1590/0074-02760150470

**Published:** 2016-05

**Authors:** Anthony J Cornel, Katherine K Brisco, Wanderli P Tadei, Nágila FC Secundino, Miriam S Rafael, Allan KR Galardo, Jansen F Medeiros, Felipe AC Pessoa, Claudia M Ríos-Velásquez, Yoosook Lee, Paulo FP Pimenta, Gregory C Lanzaro

**Affiliations:** 1 University of California, Department of Entomology and Nematology, Mosquito Control Research Laboratory, Davis, CA, USA; 2 Instituto Nacional de Pesquisas da Amazônia, Laboratório de Malária e Dengue, Manaus, AM, Brasil; 3 Fundação Oswaldo Cruz, Instituto Leônidas & Maria Deane, Laboratório de Ecologia de Doenças Infecciosas na Amazônia, Manaus, AM, Brasil; 4 University of California, Department of Pathology and Microbiology, Vector Genetics Laboratory, Davis, CA, USA; 5 Fundação Oswaldo Cruz, Centro de Pesquisas René Rachou, Laboratório de Entomologia Médica, Belo Horizonte, MG, Brasil; 6 Instituto de Pesquisas Científicas e Tecnológicas, Laboratório de Entomologia Médica, Macapá, AP, Brasil; 7 Fundação Oswaldo Cruz, Laboratório de Entomologia, Porto Velho, RO, Brasil; 8 Fundação de Medicina Tropical Dr Heitor Vieira Dourado, Manaus, AM, Brasil

**Keywords:** Anopheles darlingi, salivary gland polytene chromosomes, chromosome inversions

## Abstract

Salivary gland polytene chromosomes of 4th instar *Anopheles darlingi* Root were examined from multiple locations in the Brazilian Amazon. Minor modifications were made to existing polytene photomaps. These included changes to the breakpoint positions of several previously described paracentric inversions and descriptions of four new paracentric inversions, two on the right arm of chromosome 3 and two on the left arm of chromosome 3 that were found in multiple locations. A total of 18 inversions on the X (n = 1) chromosome, chromosome 2 (n = 7) and 3 (n = 11) were scored for 83 individuals from Manaus, Macapá and Porto Velho municipalities. The frequency of 2Ra inversion karyotypes in Manaus shows significant deficiency of heterozygotes (p < 0.0009). No significant linkage disequilibrium was found between inversions on chromosome 2 and 3. We hypothesize that at least two sympatric subpopulations exist within the *An. darlingi* population at Manaus based on inversion frequencies.

Malaria is a persistent problem in South America, with 350,000 confirmed cases reported in 2013, of these 51% were in Brazil (WHO 2014). Most malaria in Brazil (82%) is due to infection with *Plasmodium vivax* transmitted by its major vector, *Anopheles darlingi* (WHO 2014, Pimenta et al. 2015). Currently, laboratory based studies of malaria transmission are difficult because there is no reliable protocol for *P. vivax* culture (Noulin et al. 2013) and no permanent Brazilian *An. darlingi* colonies exist (Moreno et al. 2014, Pimenta et al. 2015). However, colonies of *An. darlingi* from specimens originating from the villages of Cahuide and Zungarococha in the Peruvian Amazon Region currently exist (Moreno et al. 2014, Villarreal-Treviño et al. 2015). Given the large geographic range of *An. darlingi* and the ecological diversity in Brazil and South America, we expect that this species is comprised of multiple, genetically distinct populations over its range, which single nucleotide polymorphic markers seem to suggest (Emerson et al. 2015). *Anopheles* mosquitoes cytogenetic studies of chromosome inversions have provided a foundation upon which population genetics studies of species within the genus have been based (Kitzmiller 1963, 1976).

Chromosome inversions represent a powerful evolutionary force largely due to the fact that in the heterozygous condition they suppress recombination (Rieseberg 2001, Butlin 2005). This facilitates the formation of coadapted gene complexes associated with multi-genic phenotypes that include ecological adaptations (Besansky et al. 2003) and behavioral patterns associated with mating (Herrera et al. 2014). Recent population genomics studies have revitalized interest in inversions by revealing that their occurrence in genomes is far more common than ever imagined (Feuk et al. 2005). Therefore, contemporary population genomics research must include an awareness of the presence of inversions within the genomes under study. In this paper, we present an update on the polytene chromosome map of *An. darlingi* and some preliminary data on the distribution of inversions among populations in Brazil.

The non-random distribution of inversions within the genome and a non-random distribution of inversions among geographical populations of *An. darlingi* suggest that at least some inversions are adaptive. For example in African malaria vectors in the *Anopheles gambiae* complex, there is strong evidence that certain inversions are adaptations that allowed for ecological expansions into the different environments occupied by various members of the complex (Coluzzi et al. 2002).

Populations within the Amazon Basin near the city of Manaus (Brazil) have higher inversion polymorphism compared to those in the southern regions of Brazil which are monomorphic for inversions 2Ra and 2La (Kreutzer et al. 1972). Charlwood (Charlwood 1996) noted behavioral differences within *An. darlingi* are associated with chromosome inversions and suggested that inversions may contain genes contributing to this behavioral divergence. In forested areas near Manaus, *An. darlingi* populations are primarily exophagic and exophilic, and exhibit a high degree of inversion polymorphism (Kreutzer et al. 1972, Rafael et al. 2010). In the northern region, *An. darlingi* is more endophagic and less diverse chromosomally. At the southern end of its distribution (Dourado), *An. darlingi* populations have no chromosomal polymorphism and are primarily zoophilic and exophagic.

It is to be expected that detailed and accurate chromosome maps will be needed for future studies identifying associations of chromosome polymorphisms to specific genotypes (Mirabello et al. 2008) and subpopulations (Conn et al. 2006, Angella et al. 2014) of *An. darlingi.* Detailed knowledge of polytene chromosome arrangements will also be useful for accurate genome assembly using *in situ* hybridizations of sequenced contigs (Marinotti et al. 2013) and for examining the intrinsic malaria parasite vector relations and behavioral, morphological and ecological characteristics of *An. darlingi.*

In this study, we used unstained preparations (which provide better banding resolution than stained chromosomes) to make small modifications to the existing *An. darlingi* photomap published by Rafael et al. (2010). We also discuss other specific variations in banding morphology that we found. Images of previously described (Kreutzer et al. 1972, Rafael et al. 2010) and new paracentric inversions are also provided for comparison. Preliminary results of population genetic analysis on inversion polymorphism of Manaus populations are discussed.

## MATERIALS AND METHODS

**Fig. 1:**
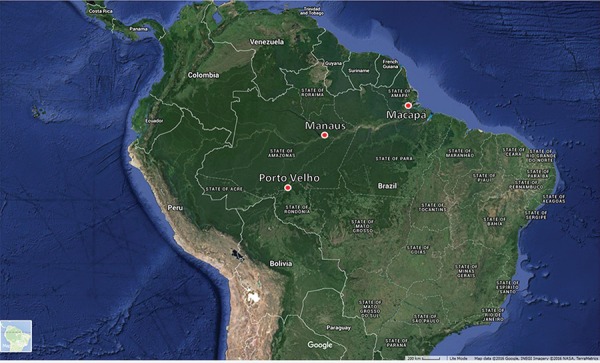
: a map of collection sites of *Anopheles darlingi* larvae, overlaid with Landstat satellite imagery sourced from Google Earth - Google Maps. ©2016 Google, INEGI Imagery and ©2016 NASA, TerraMetrics.

Salivary glands were dissected from mid-4th stage wild *An. darlingi* larvae collected from multiple locations within the Brazilian Amazon Basin (Fig. 1) in Porto Velho Municipality, Rondônia state (8.7619° S, 63.9039° W), Macapá, Amapá state (0.0339° N, 51.3500° W) and Manaus, Amazonas state (3.1000° S, 60.0167° W). Larvae from Porto Velho were collected in the last week of August 2014, followed by collections in Manaus in the first two weeks of September 2014, and followed by collections in Macapá in the third week of September 2014. Larvae were identified to species using dichotomous keys (Faran & Linthicum 1981). Methods for making chromosome preparations followed protocols described in Cornel and Collins (2000). Briefly, salivary glands were dissected in 5% propionic acid. Dissected salivary glands were transferred onto a siliconized microscope slide and a drop of modified Carnoy’s solution (one part glacial acetic acid and three parts 100% ethanol) was added to fix the tissue. Two drops of 50% propionic acid were then added before covering the tissue with a coverslip. Excess propionic acid was absorbed onto filter paper. Chromosome spreads were obtained by tapping with a pencil on the coverslip several times. We found that the best chromosome banding resolution was obtained from mid-4th stage larval salivary glands. Preparations from larvae where pupal trumpet development could be seen under the larval exoskeleton (late 4th stage) yielded chromosomes of poor banding resolution and integrity. All chromosome spread images were taken at 400x magnification using a digital camera connected to a phase contrast Leica compound microscope. Karyotype designations for each specimen examined accompanied by raw chromosome images are available in the PopI OpenProject *Adar* BR (http://popi.ucdavis.edu/). Chromosome band division numbering and subdivision lettering followed the scheme described by Rafael et al. (2010). Many of the inversion breakpoint locations represent probable locations. Accurate determination of breakpoints can only be made from inversion homozygotes. In our collections, homozygotes of many of the inversions were not obtained. None of the images of chromosome spreads in this publication were modified, ensuring natural and realistic views for interested readers. The chromosome images compared and aligned with the previously published photomaps (Rafael et al. 2010) were selected to represent the qualities of typical spreads to keep in line with our attempts to provide a more realistic representation of the chromosomes.

Guo’s Exact Hardy-Weinberg equilibrium test (HW) and Linkage Disequilibrium (LD) p values between inversions on the same and different chromosome arms were calculated using the Arlequin program (Excoffier & Lis- cher 2010). LD were calculated using the likelihood test based on Slatkin (1994) and Slatkin and Excoffier (1996).

## RESULTS AND DISCUSSION

**Fig. 2:**
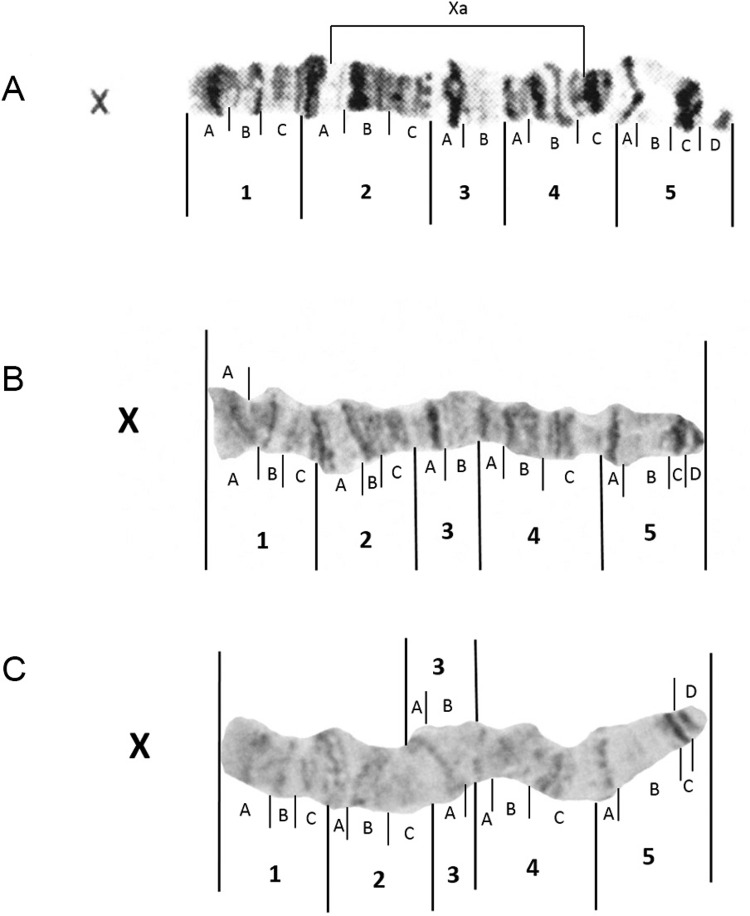
: photomap (A) and images of *Anopheles darlingi* X polytene chromosomes from Manaus, Amazonas (B) and Macapá, Amapá (C).

*X chromosome* - Images of two unstained X chromosomes in Fig. 2B (Manaus) and Fig. 2C (Macapá) show that banding intensity varies considerably between spreads. The image from Macapá (Fig. 2C) is in fact a more typical representation of faint unstained bands. No X a/+ or X a/a karyotypes were seen in larvae dissected in this study. No modifications were proposed to be made to the previously published photomap in Fig. 2A.

**Fig. 3:**
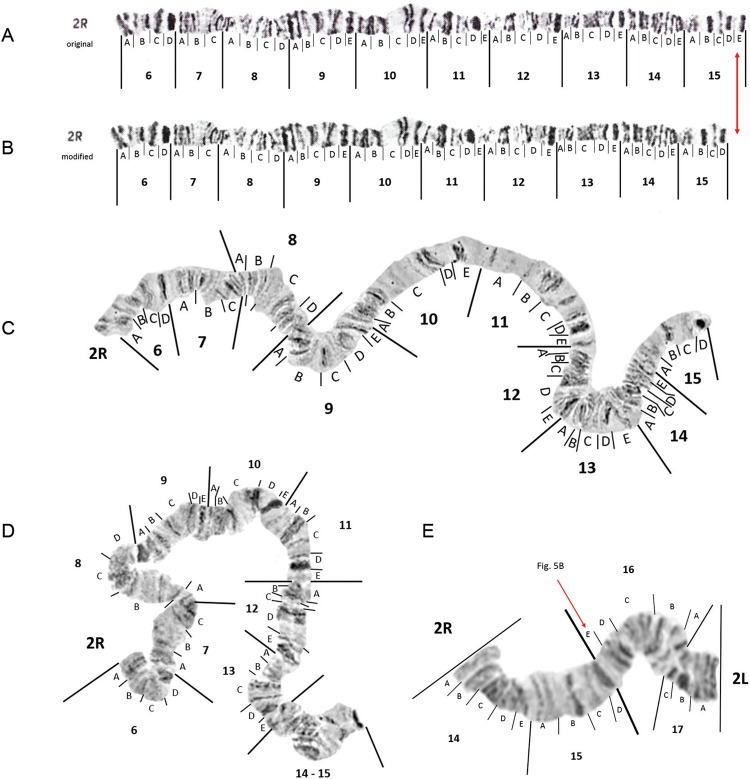
: original (insert A) and modified photomap (insert B) and images of *Anopheles darlingi* 2R polytene chromosomes from Macapá, Amapá (inserts C and D). Insert E represents the centromeric joint between 2R and 2L in a larval spread from Macapá to confirm that there is no broad band in 15E, as represented in the original photomap. Presence of two very faint bands in this region is more representative, which has been added to 2L as subdivision 16E (see Fig. 5B).

*The right arm of chromosome 2 (2R)* - The single dark band within subdivision 15E (Fig. 3A) was never seen in any of our chromosome spreads. Therefore, we have removed 15E from the original photomap as depicted in Fig. 3B.

**Fig. 4:**
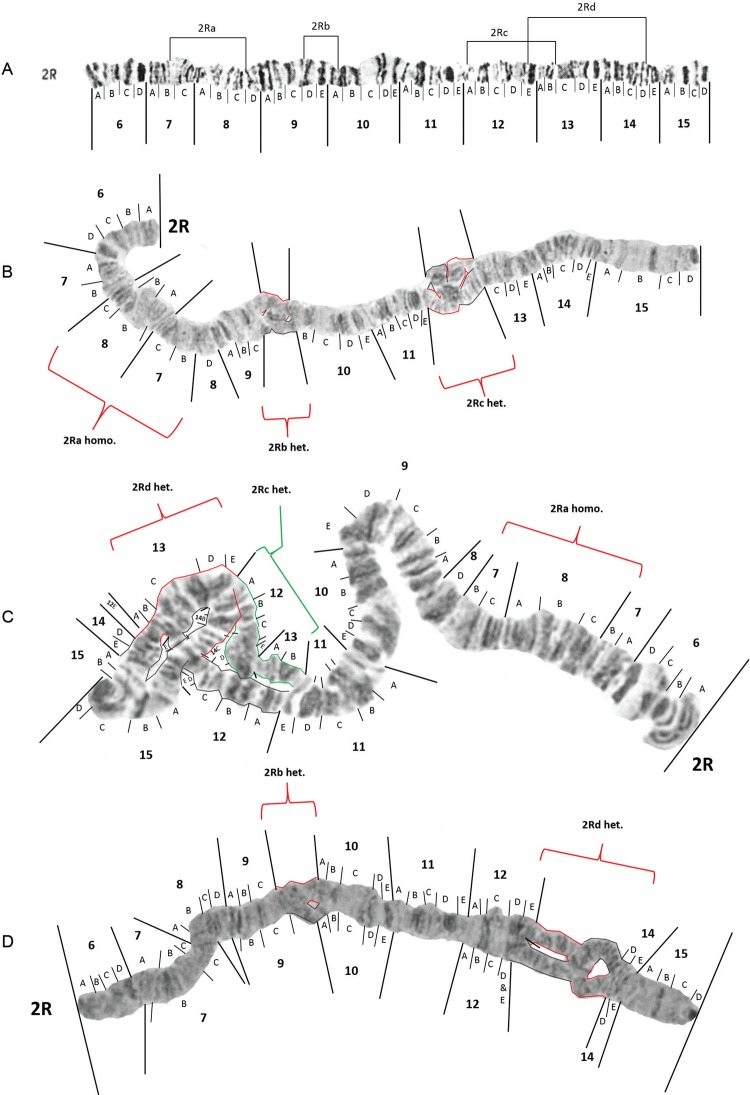
: modified photomap (insert A) and inversion arrangements of *Anopheles darlingi* 2R chromosome arm. Breakpoint positions are demarcated as lines in the photomap (insert A). Insert B is a chromosome from Manaus, Amazonas, that is homozygous for 2Ra, heterozygous for 2Rb (2Rb/+) and 2Rc (2Rc/+). Insert C is a chromosome from Manaus that is also homozygous for 2Ra and heterozygous for 2Rc, but is heterozygous for 2Rd and insert D is a chromosome from Manaus that is heterozygous for 2Rb and 2Rd.

Probable inversion breakpoints of 2Ra, 2Rb, 2Rc and 2Rd which are inversions originally described by Kreutzer et al. (1972) and Tadei et al. (1982) are shown in Fig. 4A. The centromeric breakpoint end of 2Rc has been shifted from within 12E to 13B/C based on careful examination of multiple spreads that were heterozygous for this inversion (see Fig. 4B). 2Ra homozygotes (Fig. 4B-C) were observed in 29.2% of the larvae from Manaus (Table). One 2R a/+ was seen in Manaus. Inversion 2R b/+ (Fig. 4D) occurred in 21.5% of mosquitoes sampled from Manaus and one mosquito from Porto Velho (Table). Heterozygotes of inversions 2Rc (Fig. 4B-C) were found in 46.2% of individuals from Manaus and one mosquito from Porto Velho. Heterozygotes for 2Rd (Fig. 4C-D) were seen in 27.7% of the mosquitoes in Manaus. No homozygotes of 2Rb, 2Rc or 2Rd were observed. Multiple mosquitoes from Manaus were heterozygous for two or more inversions on the same chromosome arm.

**TABLE:** Inversion frequencies in *Anopheles darlingi* found within each location

Chromosome arm	Inversion	*Anopheles darlingi* populations														

		Porto Velho					Manaus					Macapá				
		
		+/+	+/-	-/-	Total	HWE p-value	+/+	+/-	-/-	Total	HWE p-value	+/+	+/-	-/-	Total	HWE p-value
X	Xa	17	0	0	17	-	63	0	0	63	-	17	0	0	17	-
2R	2Ra	21	0	0	21	-	45	1	19	65	0.00*	17	0	0	17	-
	2Rb	20	1	0	21	1	51	14	0	65	1	17	0	0	17	-
	2Rc	20	1	0	21	1	35	30	0	65	0.02	17	0	0	17	-
	2Rd	21	0	0	21	-	47	18	0	65	0.59	17	0	0	17	-
2L	2La	21	1	0	22	1	33	33	0	66	0.01	17	0	0	17	-
	2Lb	22	0	0	22	-	66	0	0	66	-	17	0	0	17	-
	2L’	21	0	1	22	0.08	55	3	7	65	0	17	0	0	17	-
3R	3Ra	21	0	0	21	-	52	14	0	66	1	17	0	0	17	-
	3Rb	21	0	0	21	-	66	0	0	66	-	17	0	0	17	-
	3Rc	21	0	0	21	-	60	6	0	66	1	13	4	0	17	1
	3Rd	21	0	0	21	-	66	0	0	66	-	17	0	0	17	-
	3Re	20	1	0	21	-	64	2	0	66	1	17	0	0	17	1
	3Rf	21	0	0	21	-	54	11	1	66	0.48	17	0	0	17	-
3L	3La	20	1	0	21	-	59	7	0	66	1	16	1	0	17	1
	3Lb	20	1	0	21	1	56	10	0	66	1	14	3	0	17	1
	3Lc	21	0	0	21	-	65	1	0	66	1	17	0	0	17	-
	3Ld	15	6	0	21	1	45	21	0	66	0.35	14	3	0	17	1
	3Le	21	0	0	21	-	61	5	0	66	1	17	0	0	17	-

+: denotes standard arrangement; -: denotes inverted arrangement; HWE p-value: denotes p-value from Guo’s exact Hardy-Weinberg equilibrium test. Only 2Ra in Manaus was significant.

**Fig. 5:**
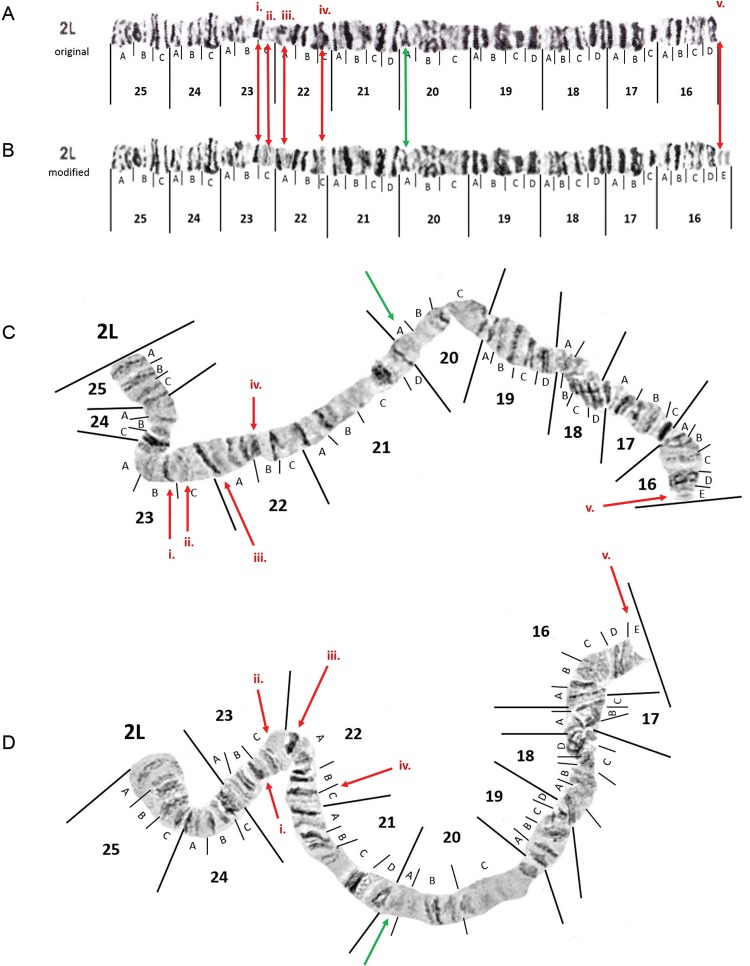
: original photomap (insert A) and modified photomap (insert B) and images of *Anopheles darlingi* 2L polytene chromosomes are depicted from Macapá, Amapá, as inserts C and D. See text for explanation of banding morphology modifications made to the original photomap that correspond to the red and green lines.

**Fig. 6:**
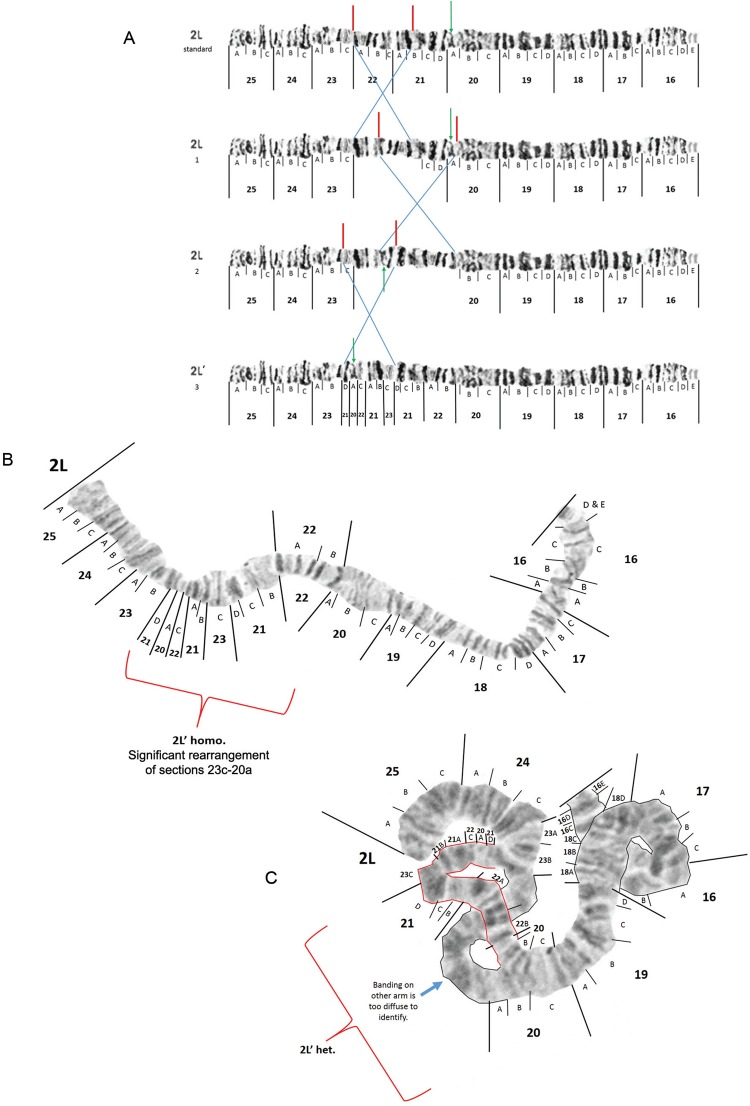
: images of the *Anopheles darlingi* 2L’ chromosome arm. Postulated inversions required to produce the hypothetical, intermediate arrangements between standard 2L and the rearranged 2L’ configuration are shown using the modified photomap in insert A. An image of a homozygote 2L’/2L’ configuration from Manaus, Amazonas, is provided in insert B and the individual from site 8 that was 2L’/? is inserted as insert C.

*Chromosome arm 2L* - Two small light colored bands were seen in almost all spreads (Fig. 5). Therefore these two small light bands have been inserted as subdivision 16E on the left arm of chromosome 2 as depicted by a red marking (v.) in Fig. 5B. In most of the spreads we examined, the two bands in 20A were lighter than as presented in the original photomap (green lines in Figs 5-6). The area in 24C is also not always as puffed as depicted in the photomap (Fig. 5C-D).

Additional modifications to the original 2L photomap (Fig. 5A) by Rafael et al. (2010) were proposed, to resemble the typical banding morphology observed in chromosome spreads from all locations we examined. We (i) removed one dark band in subdivision 23C, (ii) replaced the two very faint bands in 23C with three narrow sharp bands and broadened the boundary between 23C and 22A, (iii) replaced the distal diffuse area in 22A with one distinct dark and one distinct pale band, and (iv) lightened the dark band in 22C. These modifications were depicted in red markings in Fig. 5. We used this modified photomap to identify karyotypes in this study.

An alternative arrangement in 2L extending from regions 23C to 20A, which we have called 2L’ was seen in one specimen from Porto Velho and ten specimens from Manaus (Fig. 6B). Three specimens from Manaus (sites 8 and 9) were found in the heterozygous state, the best representation being the specimen from site 8 (Fig. 6C). We have designated this region as 2L’ rather than typical alphabet designations because the 2L’ arrangement cannot be simply derived from the standard banding configuration by fixation of inversions 2La and 2Lb as originally mentioned in Kreutzer et al. (1972). In our opinion, it involves a more complex rearrangement of three inversions as depicted in Fig. 6A. Unfortunately, we cannot identify the karyotype of the chromatid the 2L’ paired with in the heterozygous specimen (Fig. 6C) because the banding was too diffuse for a non-subjective interpretation.

**Fig. 7:**
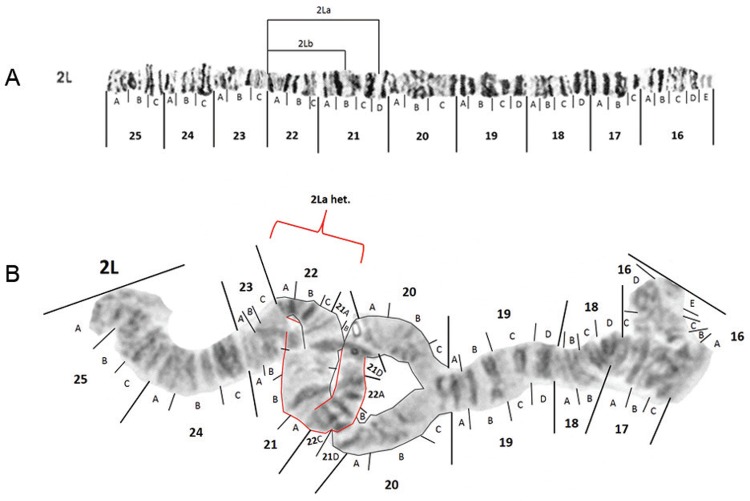
: modified photomap and inversion arrangements of *Anopheles darlingi* 2L chromosome arm. Breakpoint positions are demarcated as lines in the modified photomap (insert A). An example of a 2La/+ heterozygote is presented in insert B.

Likely breakpoints of inversions found in 2L (Kreutzer et al. 1972) are demarcated in Fig. 7A. However, according to our observations, the centromeric breakpoint of inversion 2La should be shifted from its original position in subdivision 20A (Rafael et al. 2010) to 21D. Heterozygotes for 2La (Fig. 7B) were seen in multiple mosquitoes from Porto Velho and Manaus. No specimens with inversion 2Lb were seen in our collection.

**Fig. 8:**
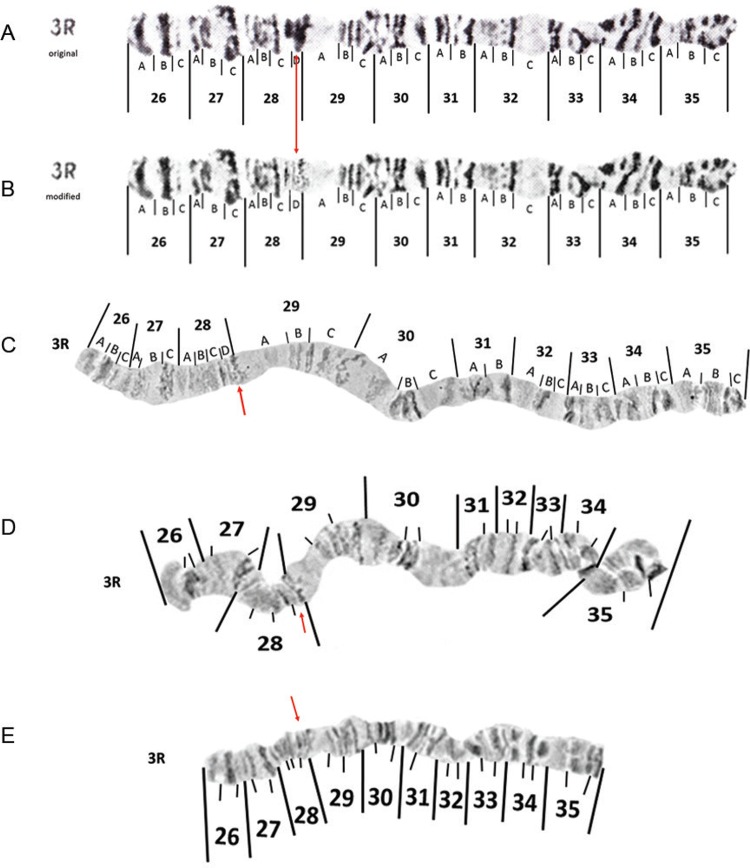
: original (insert A) and modified (insert B) photomaps and images of *Anopheles darlingi* 3R polytene chromosomes (inserts C, D and E). See text for explanation of modifications corresponding to red arrows.

*The right arm of chromosome 3 (3R)* - Changing the intensity and size of the three bands in 28C and 28D is the only change that we have proposed to make to the original Rafael et al. (2010) photomap (Fig. 8A-B). However, it should be noted that banding within divisions 34 and 35 can be variable. The banding morphology of 3R depicted in images of chromosomes as inserts C, D and E in Fig. 8 correspond to more typical banding patterns.

**Fig. 9:**
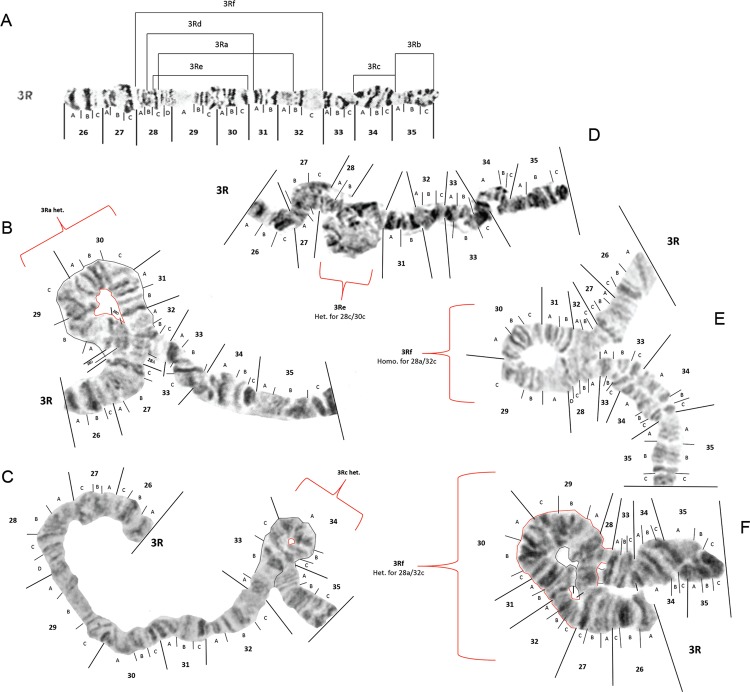
: photomap and inversion arrangements of *Anopheles darlingi* 3R chromosome arm. Breakpoint positions are demarcated as lines in the photomap (A). Examples of mosquitoes that had chromosome configurations heterozygous for inversions 3Ra, 3Rc, 3Re and 3Rf are inserted as images in B, C, D and F. An individual that was homozygous for 3Rf is shown in insert E. Images of chromosomes in inserts B, D, E and F were made from larvae collected in Manaus, Amazonas, and the one in insert C was from a mosquito collected in the site near Macapá, Amapá.

Probable inversion breakpoints identified in 3R are demarcated in Fig. 9A. Four of the inversions, *viz*. 3Ra, 3Rb, 3Rc and 3Rd, have been previously described (Kreutzer et al. 1972, Rafael et al. 2010) and two of the inversions, *viz*. 3Re and 3Rf, are newly described in this paper. Breakpoints of inversions 3Rb, 3Rc and 3Rd remain unchanged. However, after careful examination of multiple mosquitoes that were heterozygous for 3Ra, we have recommended moving both breakpoints to new locations. Half of the specimens from Manaus subsites 7 and 9 were heterozygous for inversion 3Ra (3Ra/+), as shown in Fig. 9B. A few larvae from Manaus and Macapá were 3Rc/+ (Fig. 9C). Inversions 3Rb and 3Rd were not seen in any specimens examined in this study. However, one specimen from the Porto Velho colony and two from Manaus were heterozygous for inversion 3Re (3Re/+), as shown in Fig. 9D. 3Re has breakpoints very close to 3Rd breakpoints and we are in fact unsure if this is a new inversion. A homozygote for 3Re was not observed to verify this but for now we have called this a new inversion. One new inversion which we have named 3Rf was seen in the heterozygous arrangement (3Rf/+) in three specimens from Manaus (Fig. 9F) and as a homozygote in one specimen from Manaus (Fig. 9E).

**Fig. 10:**
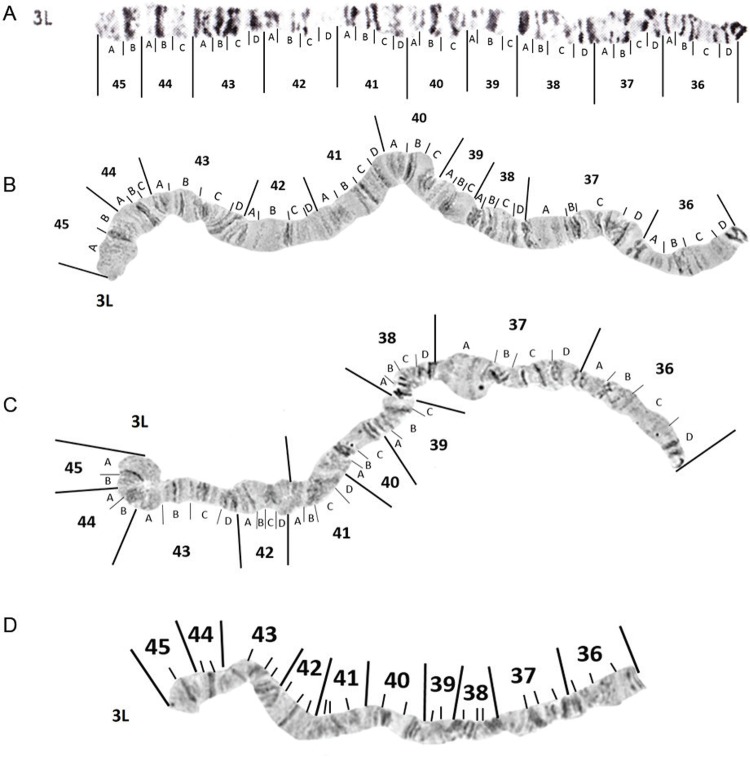
: photomap (A) and images of *Anopheles darlingi* 3L polytene chromosomes.

**Fig. 11:**
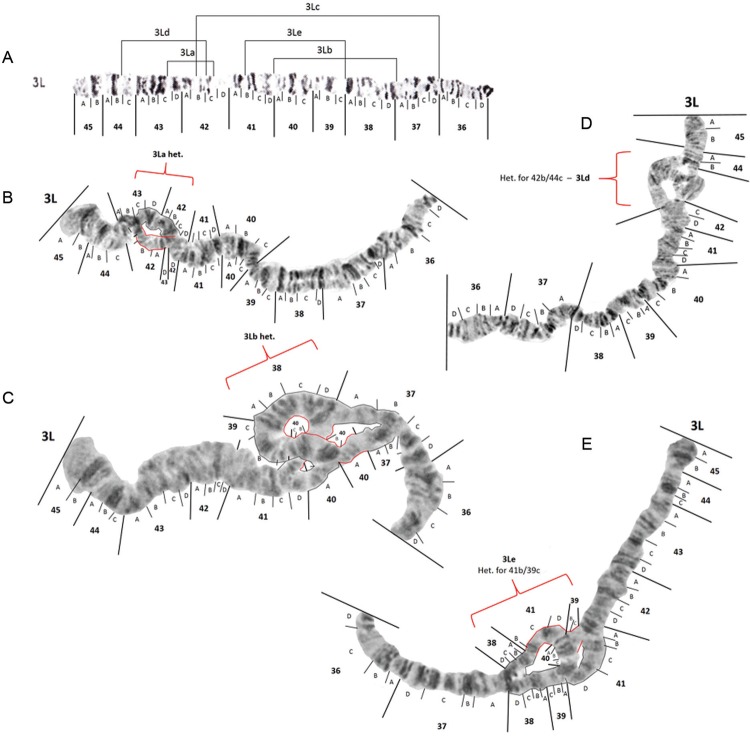
: photomap and inversion arrangements of *Anopheles darlingi* 3L chromosome arm. Inversion breakpoint positions are demarcated as lines in insert A, and examples of chromosomes dissected from mosquitoes in Manaus, Amazonas, that were heterozygous for inversions 3La, 3Lb, 3Ld and 3Le are inserted as inserts B, C, D and E respectively.

*The left arm of chromosome 3 (3L)* - No changes to the 3L photomap (Fig. 10A) were proposed. Likely inversion breakpoints found on chromosome 3L are demarcated in Fig. 11A. Three of these inversions, *viz*. 3La, 3Lb and 3Lc, have been previously described (Kreutzer et al. 1972, Rafael et al. 2010). Breakpoint positions of inversions 3La, 3Lb and 3Lc remain unchanged. Inversion 3Lc was not observed in any mosquito we examined. Heterozygotes for 3Lb (3Lb/+) (Fig. 11C) were observed in one specimen from Porto Velho, three mosquitoes from Macapá, and 10 mosquitoes from Manaus. Inversion 3La (Fig. 11B) was seen in a heterozygous arrangement (3La/+) in 10.6% of the mosquitoes from Manaus and one mosquito from both Porto Velho and Macapá. A further two new inversions *viz*. 3Ld and 3Le, were found in *An. darlingi* examined, in this study. Inversion 3Ld heterozygotes (3Ld/+) (Fig. 11D) were common, occurring in 31.8% of the larvae from Manaus, 28.6% from Porto Velho, and 17.6% from Macapá. Heterozygotes for inversion 3Le (Fig. 11E) were found in five larvae from Manaus.

**Fig. 12:**
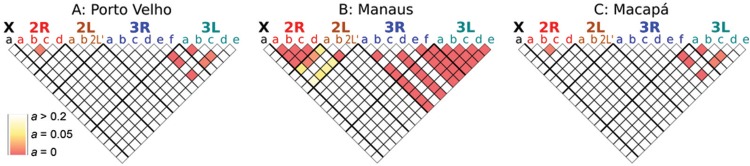
: Linkage Disequilibrium between inversions. Colors in each square denote p-value after multiple comparison adjustments (*a*). An *a* less than 0.05 indicates significant LD between inversion pairs of *Anopheles darlingi* polytene chromosomes from Manaus, Amazonas, Porto Velho, Rondônia and Macapá, Amapá.

*Inversion frequencies* - Frequencies for each inversion from the three main locations sampled (Porto Velho, Manaus and Macapá) are provided in Table. No departures from HW were found in any inversion frequencies, except for inversion 2Ra in Manaus where there was significant deficit of 2Ra/+ heterozygotes (p < 0.0009) which may be indicative of local population structure. All inversions in chromosome 2 had significant LD among each other except for 2Lb (p < 0.0003) (Fig. 12). All inversions in chromosome 3 were in LD with one another, except 3Rb and 3Rd (p = 0.000) (Fig. 12). No inversions between chromosome arms were in LD. The high degree of inversion heterozygosity in Manaus is commensurate with findings from others (Kreutzer et al. 1972, Rafael et al. 2010), especially when compared to populations in more southern and south-eastern locations of Brazil. In this study, considerably fewer inversion polymorphisms were found in *An. darlingi* south (Porto Velho) and east (Macapá) of Manaus. According to Morrone’s (Morrone 2014) bio-geographical classification, Macapá is located within the Boreal Brazilian dominion, Porto Velho is within the South Brazilian dominion and Manaus is located at the boundary between these two dominions. It is not known if the intermediate biogeographical location of Manaus has any bearing on the high inversion polymorphisms found there.

The high degree of inversion polymorphisms is interesting. No obvious phenotypic effect of the high degree of inversion polymorphisms in *An. darlingi*, especially populations within Manaus, has been found yet. There may be inversion polymorphism advantages related to balancing selection and adaptations to xenobiotic tolerances, climatic conditions, micro-organism exposure and larval competition that are unique to local Manaus and the surrounding Amazon forest region. Vittor et al. (2009) postulated that deforestation, which changes availability of shade, vegetation, hydrology, and exposure to chemical runoff from agriculture, cities and towns all likely played a significant contributory factor to the spread of *An. darlingi* in South America. Under dominance of inversions in drier savannah versus over dominance of inversions in moist highland environments in *Anopheles funestus* in Cameroon (Ayala et al. 2013) seems to parallel that observed in *An. darlingi* which also has over dominance of inversions in the moist tropical environs in Brazil. Evidence of chromosome inversion arrangements facilitating local environmental adaptations and spread into new locations have been found in other insects, such as fruit flies (Rane et al. 2015).

Examination of inversion polymorphisms and frequencies across the entire range of *An. darlingi* will be an essential next step, especially when examined in the context of *An. darlingi* potentially consisting of three genetic clusters and perhaps species as hypothesized by Emerson et al. (2015). Examination of inversion polymorphisms in the two current *An. darlingi* colonies will also be useful. If homozygotes of inversions can be selected for in the colonies it would aid in more accurately pinpointing the breakpoint positions of the inversions. Most inversions have only been seen in the heterozygote arrangements which have thus far made identifying the breakpoint positions subjective.
